# Gene expression patterns unveil a new level of molecular heterogeneity in colorectal cancer

**DOI:** 10.1002/path.4212

**Published:** 2013-07-08

**Authors:** Eva Budinska, Vlad Popovici, Sabine Tejpar, Giovanni D'Ario, Nicolas Lapique, Katarzyna Otylia Sikora, Antonio Fabio Di Narzo, Pu Yan, John Graeme Hodgson, Scott Weinrich, Fred Bosman, Arnaud Roth, Mauro Delorenzi

**Affiliations:** 1 Bioinformatics Core Facility, Swiss Institute of Bioinformatics (SIB)Lausanne, 1015, Switzerland; 2 Institute of Biostatistics and Analyses, Masaryk UniversityBrno, Czech Republic; 3 Department of Oncology, University Hospital Gasthuisberg, Katholik Universiteit LeuvenBelgium; 4 University Institute of Pathology, Lausanne University Medical CentreSwitzerland; 5 Pfizer Inc., Worldwide Research and Development, Oncology Research UnitLa Jolla, CA, USA; 6 Oncosurgery, Geneva University HospitalSwitzerland; 7 Swiss Group for Clinical Cancer Research (SAKK)Bern, Switzerland; 8 Département de Formation et Recherche, Lausanne University Medical CentreSwitzerland

**Keywords:** colorectal cancer, histopathology, gene expression, molecular heterogeneity

## Abstract

The recognition that colorectal cancer (CRC) is a heterogeneous disease in terms of clinical behaviour and response to therapy translates into an urgent need for robust molecular disease subclassifiers that can explain this heterogeneity beyond current parameters (MSI, *KRAS*, *BRAF*). Attempts to fill this gap are emerging. The Cancer Genome Atlas (TGCA) reported two main CRC groups, based on the incidence and spectrum of mutated genes, and another paper reported an EMT expression signature defined subgroup. We performed a prior free analysis of CRC heterogeneity on 1113 CRC gene expression profiles and confronted our findings to established molecular determinants and clinical, histopathological and survival data. Unsupervised clustering based on gene modules allowed us to distinguish at least five different gene expression CRC subtypes, which we call surface crypt-like, lower crypt-like, CIMP-H-like, mesenchymal and mixed. A gene set enrichment analysis combined with literature search of gene module members identified distinct biological motifs in different subtypes. The subtypes, which were not derived based on outcome, nonetheless showed differences in prognosis. Known gene copy number variations and mutations in key cancer-associated genes differed between subtypes, but the subtypes provided molecular information beyond that contained in these variables. Morphological features significantly differed between subtypes. The objective existence of the subtypes and their clinical and molecular characteristics were validated in an independent set of 720 CRC expression profiles. Our subtypes provide a novel perspective on the heterogeneity of CRC. The proposed subtypes should be further explored retrospectively on existing clinical trial datasets and, when sufficiently robust, be prospectively assessed for clinical relevance in terms of prognosis and treatment response predictive capacity. Original microarray data were uploaded to the ArrayExpress database (http://www.ebi.ac.uk/arrayexpress/) under Accession Nos E-MTAB-990 and E-MTAB-1026.

## Introduction

Current classifications of sporadic colorectal cancer take into consideration stage, histological type and grade 1. Colorectal cancer (CRC) is a highly heterogeneous disease, with clinicopathologically similar tumours differing strikingly in treatment response and patient survival. These differences are only partly explained by current concepts regarding the molecular events leading to CRC. In recent years, microsatellite instability (MSI) emerged as an important classifier with significant prognostic impact and potential for patient stratification for therapy 2—3. Some molecular markers, as well as the mutation status of *BRAF* or *KRAS* genes (predictive for anti-EGFR 4), are in use for treatment decisions and patient stratification. However, patient groups defined by these molecular markers still differ remarkably in behaviour and therapy response 5—6. Several approaches to further subtype CRC have been proposed, based on combinations of clinical, histopathological, gene expression, CNV, epigenetic and single gene parameters 7—13. Each of these different modalities provides its own perspective on the same underlying biological reality. The CpG island methylator phenotype (CIMP) status is emerging as important molecular determinant of CRC heterogeneity 11. The cancer genome atlas (TCGA) analysis identified a hypermutant group not entirely captured by MSI status 13. Several studies have addressed CRC subtyping using genome-wide gene expression profiling of relatively large patient cohorts 12—14. One study used unsupervised clustering of stage II and III CRCs to identify three stage-independent subtypes, with *BRAF* mutation and MSI status dominating one of the subtypes 14. A study of stage I–IV CRC samples segregated CRC into two prognostic subtypes with epithelial–mesenchymal transition (EMT) as a main determinant 12. Another study on 88 stage I–IV samples identified four subtypes, one correlated with MSI, *BRAF* mutation and mucinous histology, two with stromal component and one with high nuclear *β*-catenin expression 15.

We recently reported CRC expressing a *BRAF*-mutated signature 6, which strongly overlaps with the methylation-based group of Hinoue 11, and a MSI-like gene expression group that captures the hypermutant tumours of TCGA 13, indicating the potential for identification of robust biological subgroups. We now describe CRC subtypes based upon unsupervised clustering of genome-wide expression patterns. We characterized these subtypes in terms of biological motifs, common clinical variables, association with known CRC molecular markers and morphological patterns. A key element in our approach was the use of a system of unsupervised gene modules—groups of genes with correlated expression. They are more resistant to noise and have a higher chance of having at least a few members represented on various platforms. In addition, as each gene module is represented by its median expression, the modules with fewer genes contribute equally to the subtype definition. We and others have successfully used similar strategies previously 16,17. We validated the existence of the subtypes and their respective clinical and molecular marker characteristics in an independent dataset. Ultimately, it will be mandatory to integrate the various sources of information on CRC heterogeneity into an integrative, robust and reproducible subclassifier that can become a tool for clinical use.

## Materials and methods

A detailed description of all the datasets and analysis procedures is given in Supplementary methods and results (see Supplementary material).

### Data acquisition and processing

We have built two non-overlapping data collections: a discovery collection, comprising four publicly available (425 samples) and two previously unpublished datasets (688 samples with 10 year follow-up in a clinical trial setting and 64 normal samples) with known stage status, and a validation collection of eight publicly available datasets (720 CRC samples) (see Supplementary material, Supplementary methods and results). Observations derived from the analysis of 64 normal samples were further validated on five publicly available datasets, with both carcinoma and normal samples available in one batch (totalling 205 normal/adenoma/carcinoma samples). Copy number data was available for 154 of the PETACC3, as in 19. Our analysis included a total of 2102 samples.

The discovery collection contained the previously unpublished 688 CRC formalin-fixed, paraffin-embedded (FFPE) samples of PETACC3 6 and 64 FFPE normal colon tissue samples from Centre Hospitalier Universitaire Vaudois's Biobank, which were uploaded to ArrayExpress (http://www.ebi.ac.uk/arrayexpress/), under Accession Nos E-MTAB-990 and E-MTAB-1026, respectively. Gene expression data were processed by standard tools to obtain normalized, probeset-level expression data. For each EntrezID in the datasets, the probeset with the highest variability was selected as representative and the number of EntrezIDs entering the analysis was reduced to 3025 by applying non-specific filtering. For PETACC3 and normal colon samples, patients signed an informed consent form in which the use of tissue specimens was included, and all marker study proposals were subjected to the approval of the trial steering committee.

### Subtype definition and validation

For model development (gene modules and subtype definition, classifier training, identification of subtype-specific genes) only the 1113 CRC samples of the discovery set were used, no sample in the validation collection being used for any model tuning. Hierarchical clustering (complete linkage, Pearson correlation similarity measure) and dynamic cut tree 20 were used to produce *gene modules* (groups of genes with correlated expression), from which non-robust modules (see Supplementary material, Supplementary methods and results) and a gender-related module were discarded. Each expression profile was then reduced to a vector of *meta-gene*s by taking the median of the values of genes in each gene module. The meta-genes were then further grouped into clusters using hierarchical clustering.

The subtypes were defined in terms of *core samples*—those samples from the discovery collection that were assigned to clusters by hierarchical clustering, using a consensus distance 21 followed by pruning of the dendrogram (see Supplementary material, Supplementary methods and results). The clusters to which the core samples were assigned were called *subtypes*. The rest of the samples from the discovery collection, not assigned to subtypes by this procedure, were called *non-core samples*. This approach allowed the reduction of noise in subtype-defining samples, and thus a higher consistency of the resulting subtypes defining the ground truth for downstream analyses. The stability of the obtained clusters was assessed under different perturbations of the processing pipeline (different parameters and clustering methods) to ensure that the results were not simple artefacts (see Supplementary material, Supplementary methods and results). A multiclass linear discriminant (LDA) 22 was trained on core samples with meta-genes as variables to assign new samples to one of the subtypes. Minimal gene sets characteristic to each subtype were identified using ElasticNet 23 on gene-level data.

In order to validate the existence of subtypes (and their independence on data selection) and the modelling choices in subtype discovery, we applied the same subtyping procedure (including parameters) to the validation collection. The clusters identified in the validation collection were put in correspondence with the subtypes in the training set by LDA predictions and correlations of subtype-specific moderated *t* statistic 24 values, corresponding to the gene-wise comparison of the respective subtype with the other subtypes (one-versus-all comparison). A simple classifier application would have led the validation samples to be classified as one of the subtypes, but it would have not informed us of possible over-fitting of the data in the discovery procedure.

### Subtype characterization

If not specified differently, all the reported *p* values were adjusted for multiple hypothesis testing, using the Benjamini–Hochberg procedure. Significance level was set at 0.1. Pathway analysis for each set of gene modules was carried out using the Database for Annotation, Visualization and Integrated Discovery (DAVID) 25. Gene set enrichment analysis of gene signatures was performed using the mygsea2 tool, in each subtype and normal samples, on average expression-ordered median-centred lists of genes. Differential expression analysis was performed using limma 24 and sign test using BSDA 26. The Cox proportional hazards model was used to analyse the prognostic value of interquartile range (IQR)-standardized values of meta-genes, for overall survival (OS), relapse-free survival (RFS) and survival after relapse (SAR), stratified by dataset. The Wald test was used to assess the global significance of the models. Pairwise differences in survival were assessed using the log-rank test. For subtype comparison, the survival was truncated at 7 years. Subtype enrichment for clinical or molecular markers was assessed by the Fisher test to the baseline, defined as the proportion of the marker in the whole dataset. Morphological pattern differences were assessed pairwise by Fisher test.

### Histology

**Figure 4:**
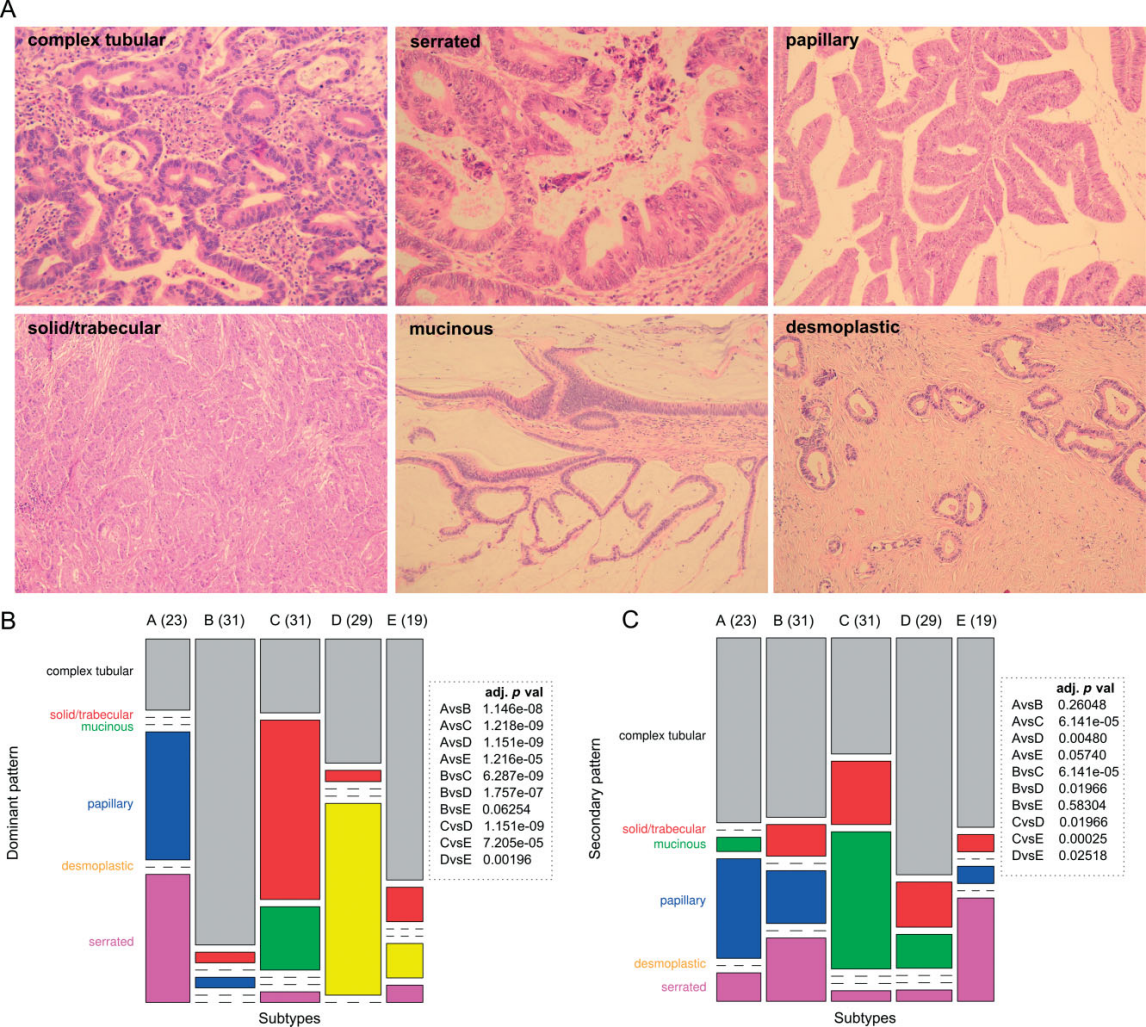
Morphological CRC patterns. (A) morphological CRC patterns scored in subtypes. (B, C) Distribution of dominant (B) and secondary (C) histological patterns in subtypes. Columns represent subtypes and widths are proportional to subtype frequency (numbers of samples in each subtype); rows represent dominant (B) or secondary (C) patterns and heights are proportional to pattern frequency. Boxes show adjusted *p* values of pairwise statistical testing of morphological pattern distribution between subtypes.

The identified subtypes were characterized histologically in terms of six different architectural patterns: complex tubular; solid/trabecular; mucinous; papillary; desmoplastic; and serrated (Figure 4A), which were called dominant or secondary depending on their presence in the histology slides (for details on immunohistochemistry, see Supplementary material, Supplementary methods and results).

## Results

### Gene modules and subtype definition

**Figure 1:**
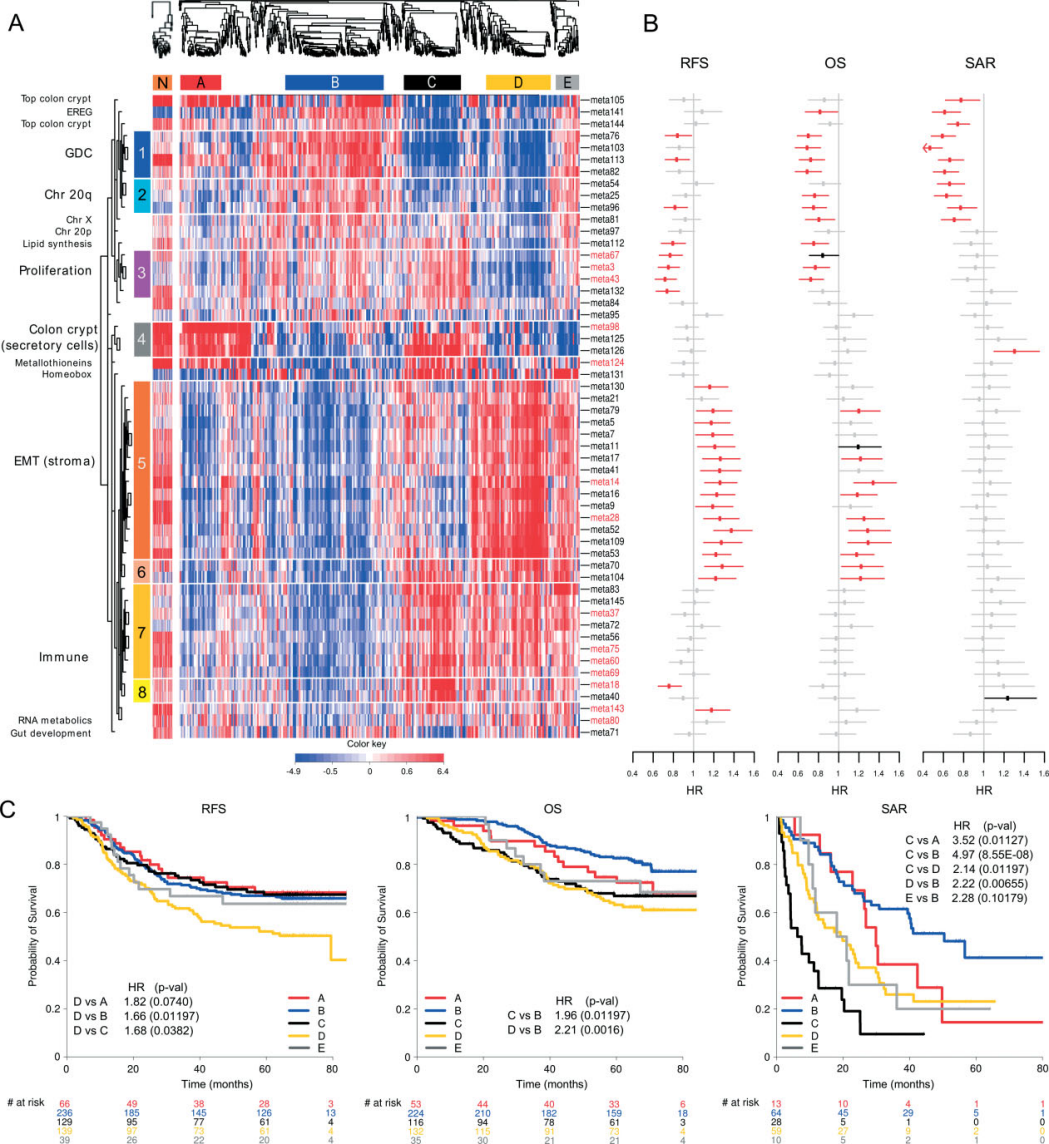
Meta-gene expression pattern in subtypes, connected with prognostic effect of subtypes and meta-genes, in the discovery set. (A) Two heat maps clustering normal (left) and CRC (right) samples (columns) and meta-genes (rows). Colours represent decreased (blue) or increased (red) meta-gene expression relative to their medians. Normal samples were clustered independently on meta-genes centred to CRC meta-gene medians. For comparative purposes, ordering of meta-genes in normal samples is imposed to correspond to that of CRC samples. White horizontal lines denote eight unsupervised clusters of meta-genes, each assigned a colour bar on the left; meta-genes not belonging to a cluster have no colour bar. Names of the meta-genes corresponding to gene modules with gene–gene correlations in normal samples comparable to those in cancer samples are marked red (see Supplementary material, Figure S1D). (B) Effect of inter-quartile range (IQR) standardized expression of meta-genes on RFS, OS and SAR. Points represent estimated hazard ratio (HR), bars represent 95% CI. Bold lines represent effects significant at 5% without adjustment for multiple hypothesis testing; red lines represent effects significant at FDR < 10%; details are provided in Table S6 (see Supplementary material). (C) Kaplan–Meier plots for RFS, OS and SAR, with HR for significant pairwise comparisons (*p* values adjusted for FDR). Numbers below *x* axes represent number of patients at risk at selected time points.

We identified 54 gene modules, reproducible across all datasets in the discovery collection, comprising 658 genes from an initial list of 3025 identified as the most variable. The assignment of genes to gene modules and gene module clusters is listed in Table S1 (see Supplementary material); meta-gene expression profiles for the discovery set are shown in Figure 1A; and between meta-gene correlations in Figure S1C (see Supplementary material). Based on gene modules, we identified five major subtypes: surface crypt-like (A), lower crypt-like (B), CIMP-H-like (C), mesenchymal (D) and mixed (E), totalling 765 samples (69% of discovery data; see Supplementary material, Supplementary methods and results).

### Subtype reproducibility in an independent validation set

In the validation set of 720 CRC samples we identified a set of subtypes comprising 602 samples (83.6% of the validation set) and associated them with our discovery subtypes using the subtype classifier (see Supplementary material, Table S2) and correlations of subtype-specific patterns based on moderated *t* statistic (see Supplementary material, Table S3). All five major subtypes reappeared in the validation set, confirming the robustness of our approach. Figure S2 (see Supplementary material) presents gene expression profiles of both discovery and validation sets. Two notable differences were observed: (i) subtype B in the validation set was split into two subgroups (B1, B2), as observed in the discovery set too, but only at lower pruning height; (ii) another cluster passed the minimal size criteria, corresponding to the small subtype (F) which, in the discovery set, was not considered for further characterization because of small sample size. Validation of other subtype characteristics (to the extent of available information) is described in each of the respective sections.

### Subtypes are characterized by distinct biological components

We set out to assign biological labels to gene modules that define the subtypes (Table 1; see also Supplementary material, Table S1). Of the 54 meta-genes, 41 could be further grouped into eight gene module clusters; 13 meta-genes remained ungrouped, each possibly representing a distinct biological motif. Pathway analysis characterized five of eight gene module clusters by the following biological motifs: chromosome 20q (cluster 2), proliferation (cluster 3), EMT/stroma (cluster 5) and immune response (clusters 7 and 8). Literature searching identified biological motifs associated with other gene modules. We labelled cluster 1 as GDC (genes differentially expressed in CRC), as it consisted of a number of genes significantly associated with CRC. The analysis of pairwise intra-gene module correlations in normal samples of both discovery and validation set identified as cancer-specific gene modules of chromosome 20q, several immune response, EMT/stroma and GDC gene modules, homeobox genes and gut development (see Supplementary material, Figure S1D). The relationship between subtypes and meta-genes is illustrated by the heat map (Figure 1A), in which the major molecular motifs and their role in subtype definition stand out. Table S4 (see Supplementary material) contains median subtype values per meta-gene and the results of differential meta-gene expression testing between subtypes. Subtypes are not determined by individual biological components but each of them contributes to the molecular identity of the subtypes. The EMT/stroma cluster stands out in subtypes A + B (low expression) and D + E (high expression), while subtype C notably contained a high expression of immunity-associated cluster. High expression of meta-genes representing upper colon crypt cells in subtypes A and B, correlated with serrated and papillary (A) and complex tubular (B) morphological patterns (see below). Given the enterocyte-like morphology and retained polarity of the neoplastic cells in these patterns, they are considered as well differentiated. Subtype C is associated with the mucinous phenotype. Interestingly, subtypes A and C show high expression of metallothioneins, subtypes C and E show high expression of the homeobox gene module, while subtypes E and B strongly express a gene module containing the *EREG* gene (Table 1). The high expression of chromosome 20q cluster in subtype B was correlated with a significantly higher copy number gain/amplification of all of 20q in this subtype (see Supplementary material, Figure S8). The low expression of lipid synthesis genes is striking for subtype D and low expression of the gut development gene module for subtype C. A refined picture of differences is given by a quantitative comparison of (meta-)gene expression between subtype pairs (see Supplementary material, Tables S4 and S5, Figure S4). For each subtype we also identified a minimum set of characteristic genes (Table 2; for more details, see Supplementary material, Supplementary methods and results).

**Table 1:** Biological identification of gene modules

Cluster name	Number of genes	Pathway analysis result (number of overlapping genes, *p* value) OR description based on literature search	Selected genes
1. GDC	27	Genes involved in differentiation of colon crypt and/or whose expression was reported to be affected in colorectal cancer and/or with prognostic effect in CRC	Intestinal differentiation genes: *CDX2*^45^, *IHH*^46^, *VAV3*^47^, *ASCL2*^35^, *PLAGL2*^48^ Genes reported altered in colorectal cancer with prognostic effect: *PITX2*^49^, *DDC*^50^, *PRLR*^51^, *SPINK1*^52^ Other genes connected to CRC: *GGH*–connected to CIMP^+^ phenotype 53 *NR1I2*–connected to chemoresistance 54
2. Chromosome 20q genes	33	Chromosome 20 (26 genes, 9.2E-34)	Other, non-20q genes: *TP53RK*, *ANO9*, *NEU1*, *CLDN3*, *PRSS8*
3. Proliferation	83	Cell cycle (36 genes, 3.0E-33) Mitosis (26 genes, 1.4E-29) Chromosome (26 genes, 2.5E-17) DNA metabolic process (20 genes, 4.9E-10) Lipid synthesis (4 genes, 5.0E-2)	Mitotic checkpoint kinases: *BUB1*, *BUB1B* Cyclins: *CCNA2*, *CCNB2* Centromere proteins: *CENPA*, *CENPE*, *CENPN* Kinesins: *KIF11*, *KIF23*, *KIF4A* Topoisomerase II (*TOP2A*) Cell division cycle 2 *CDC2*
4. Colon crypt markers (secretory cells)	16		*AGR2*^55^, *AGR3*, *MUC2*, *SPINK4*^56^, *RETNLB*^57^, *REG4*^58^
5. EMT/stroma	310	Extracellular region part (90 genes) 2.7E-36 Cell adhesion (57 genes) 1.2E-17 Extracellular matrix (44 genes) 5.3E-30 Collagen (16 genes) 1.2E-15 EGF-like domain (26 genes) 1.6E-12 Cell motion (33 genes) 7.2E-8 Blood vessel development (25 genes) 1.1E-8 Growth factor binding (6 genes) 6.0E-5 Frizzled related (5 genes) 6.7E-3 Cell junction organization (7 genes) 1.8E-2 WNT receptor signalling pathway (8 genes) 1.4E-1	Inhibitors of *β*-catenin-dependent canonical WNT: *SFRP1*, *SFRP2*, *SFRP4*, *DKK3*, *FZD1*,*7*, *PRICKLE1*, *NXN* Mesenchymal markers: N-cadherin, OB cadherin, *SPARC*, *DDR2* EMT inducers(TFs): *SNAI2*, *ZEB1*, *ZEB2*, *TWIST1*, *CDH11* ECM remodelling and invasion: *MMP14*, *VIM* ECM proteins: fibronectin 1, collagens Angiogenesis: *PLAT*, *PLAU*, *NRP1*, *NRP2*, *THBS1*, *THBS2*, *THBS4* TGFs, their receptors and binding proteins: *IGF1*, *IGFBP5*, *IGFBP7*,*TGFB*, *LTBP1*, *LTBP2*, *PDGFRA*, *PDGFRB*
6. Unidentified	14		*DUSP1*, *EGR2*, *SERPINE1*
7 and 8. Immune response	103	Immune response (42 genes) 2.0E-28 Positive regulation of immune system process (16 genes) 4.0E-9 Antigen processing and presentation via MHC class II (6 genes) 7.5E-5 Defence response (31 genes) 3.3E-17 Chemokine signalling pathway (9 genes) 2.2E-3 Lymphocyte activation (11 genes) 2.1E-5 Regulation of programmed cell death (14 genes) 2.1E-2	Cytokines: *CCL3*, *CXCL5*, *CXCL9*,*CXCL10*, *CXCL11*, *SPP1*, *LTB* MHC class II: *HLA-DMB*, *HLA-DPA1*, *HLA-DRA*, *CD74* MHC class I: *HLA-F*, *TAP1*, *TAP2* Anti-apoptotic: *BCL2A1*, *CD74*, *BIRC3*, *IFI6*, *TNFAIP3*, *TNFAIP3* Apoptotic: *STAT1*, *XAF1* Interferon-induced proteins: *IFI30*, *IFI16*, *IFI44*, *IFI16*, *IFIH1*, *IFIT3*
*Cluster-unassigned meta-genes with colon crypt cell markers (enterocytes/top of the crypt)*			
Meta-gene 105	6	Top of the crypt genes	*FAM55A*, *FAM55D*, *MUC12* and *CEACAM7*^59^, *SLC26A2*^59^, *SLC26A3*^59^
Meta-gene 144	5	Enterocytes, goblet cells markers	*LOC644844*, *NGEF*, *HEPH*, *KRT20*^59^, *MUC20*^59^
*Cluster-unassigned meta-genes associated with chromosomal location 0*			
Meta-gene 81	7	Chromosome X (7 genes) 1.1E-8	*CXorf15*, *EIF1AX*, *HDHD1A*, *MED14*, *PNPLA4*, *SCML1*, *SMC1A*
Meta-gene 97	6	Chromosome 20p (5 genes) 5.0E-11	*CDC25B*, *CSNK2A1*, *MRPS26*, *PTPRA*, *RP5-1022P6.2*, *SNRPB*
Meta-gene 84	7	Chromosome 8 (7 genes) 5.4E-9	*AGPAT5*, *FDFT1*, *GTF2E2*, *LONRF1*, *MTUS1*, *VPS37A*, *ZNF395*
*Other cluster-unassigned meta-genes*			
Meta-gene 141	5	EREG	*AK3L1*, *ARID3A*, *EREG*, *LDLRAD3*, *ZBTB10*
Meta-gene 112	6	Lipid synthesis (4 genes) 5.0E-2	*DHCR7*, *FASN*, *FGFBP1*, *HMGCS1*, *IDI1*, *PCSK9*
Meta-gene 95	6	Homeobox genes	*HOXA10*, *HOXA11*, *HOXA13*, *HOXA5*, *HOXA7*, *HOXA9*
Meta-gene 124	5	Metallothioneins	*MT1E*, *MT1F*, *MT1G*, *MT1M*, *MT1X*
Meta-gene 131	5	Disulphide bonds (5 genes) 1.7E-02	*CXCL5*, *IL6*, *MMP1*, *MMP3*, *PTGS2*
Meta-gene 143	5	Unidentified	*DUSP5*, *ERRFI1*, *KLF6*, *MXD1*, *PLAUR*
Meta-gene 80	7	Regulation of RNA metabolic process (6 genes) 4.9E-2	*ATF3*, *C8orf4*, *FOS*, *JUNB*, *NR4A1*, *SIK1*, *ZFP36*
Meta-gene 71	8	Gut development (3 genes) 3.5E-2	*CCL11*, *CH25H*, *EDNRB*, *F2RL2*, *FOXF1*, *FOXF2*, *PCDH18*, *WNT5A*

**Table 2:** Subtype-specific minimal gene set as identified by Elastic net

	Minimal gene sets specifying a subtype		
Subtype	Up-regulated from population mean	Down-regulated from population mean
A. Surface crypt-like	*ADTRP*, *B3GNT7*, *CLCA1*, *MUC2*, *NR3C2*, *PADI2*, *RETNLB*, *STYK1*	*CHI3L1*, *FNDC1*, *TIMP3*, *SULF1*
B. Lower crypt-like	*CCDC113*, *CDHR1*, *FARP1*, *GPSM2*, *GRM8*, *HNF4A*, *IHH*, *KCNK5*, *KIAA0226L*, *MYRIP*, *PLAGL2*, *PRR15*, *QPRT*, *RNF43*, *RPS6KA3*, *SLC5A6*, *TP53RK*, *TSPAN6*, *VAV3*, *YAE1D1*	*ALOX5*, *BASP1*, *CREB3L1*, *CXCR4*, *EPB41L3*, *FSCN1*, *GFPT2*, *GPX8*, *ITPRIP*, *KCNMA1*, *KCTD12*,*MT1E*, *RARRES3*, *RNASE1*, *SGK1*, *SOCS3*
C. CIMP-H-like	*ANP32E*, *EGLN3*, *IDO1*, *PLK2*, *RAB27B*, *RARRES3*, *RPL22L1*, *TFAP2A*	*ATP9A*, *C10orf99*, *CXCL14*, *KIAA0226L*
D. Mesenchymal	*ANK2*, *BOC*, *C7*, *CRYAB*, *DCHS1*, *DDR2*, *GEM*, *PRICKLE1*, *TAGLN*	*HOOK1*, *RBM47*
E. Mixed	*CEACAM6*, *CXCL5*, *HSD11B1*, *IL1B*, *IL6*, *MRPS31*, *PI15*, *RAP2A*, *UQCC*	*AGR3*, *RAB27B*, *REG4*

### Normal colon mucosa in the context of subtypes

When applied to the 64 normal samples, the LDA classifier assigned them all to subtype A, with posterior probability > 0.99, supporting the observation that A is well differentiated and closest to normal colonic epithelium in terms of gene expression pattern. For validation, we analysed five public datasets comprising 205 profiles of normal/adenoma/carcinoma samples. Most of the normal and adenoma samples were classified by LDA as subtype A (74.5% of 51 and 69.0% of 71, respectively) or subtype B (28.2% and 21.6%, respectively), confirming subtype A as the most normal-like. The 80 carcinoma samples were distributed over all subtypes (26.2% A, 30.0% B, 11.3% C, 18.7% D and 13.8% E).

### Subtypes and patient survival

We assessed whether subtypes differ in survival, as a general read-out of biological significance, and then tested the association of each meta-gene with prognosis, using the complete discovery set of 1113 patients (Figure 1B-C see also Supplementary material, Table S6). Kaplan–Meier curves for RFS, OS, SAR, hazard ratios (HRs) and *p* values of pairwise differences between subtypes are shown in Figure 1C. The results indicate that subtypes C and D are associated with poor OS. For subtype D, this is primarily due to early relapse correlated with high expression of EMT genes and low expression of proliferation-associated genes. For subtype C it is the result of short SAR, correlated with low expression of GDC, top colon crypt, EREG and Chr 20q genes and high expression of meta-gene 126 (see Supplementary material, Table S1). For subtype E the trend towards poorer OS and RFS was not statistically significant, although borderline significant poorer SAR was found relative to subtype B. Subtypes A and B had better prognosis than D for all three endpoints, although for OS in subtype A this was not significant.

The analysis of clinical and molecular markers (below) showed that subtype C is enriched for MSI tumours and *BRAF* mutant tumours, the latter present also in subtype D. The literature indicates that MSI is associated with better RFS, while *BRAF* mutation is an indicator of worse SAR 27. To analyse how these two contradictory components affect survival in subtypes, we built a multivariate Cox proportional hazard model with subtype, stage, *BRAF* and MSI (Table 3; see also Supplementary material, Table S6). Subtype C remained significantly associated with poor SAR, even after the adjustment for *BRAF*, MSI and stage, but not with RFS. Subtypes B and D remained significantly prognostic for RFS, OS and SAR. No equivalent survival data were available for the datasets in the validation series, hence these observations could not be validated.

**Table 3:** Result of additive multivariate Cox proportional hazards model, with subtype, *BRAF* mutation, MSI and stage^a^

Variable	RFS HR	*p*	OS HR	*p*	SAR HR	*p*
A	0.906	0.760	1.381	0.390	1.726	0.180
C	0.940	0.850	1.560	0.220	3.675	0.0022*
D	1.688	0.0055*	2.161	0.0011*	1.906	0.014*
E	1.506	0.210	2.201	0.035*	2.046	0.075
*BRAFm*	1.633	0.085	2.472	0.0034*	3.361	0.00072*
MSI	0.478	0.044*	0.275	0.004*	0.356	0.036*
Stage 3	0.770	0.190	0.943	0.820	1.780	0.062*

a Baseline is subtype B, MSS, *BRAF* wt and Stage 2.

* ariables significant in the model.

Hazard ratios (HR) for relapse-free survival (RFS), overall survival (OS) and survival after relapse (SAR).

### Colorectal stem cell and Wnt signatures within subtypes

**Figure 2:**
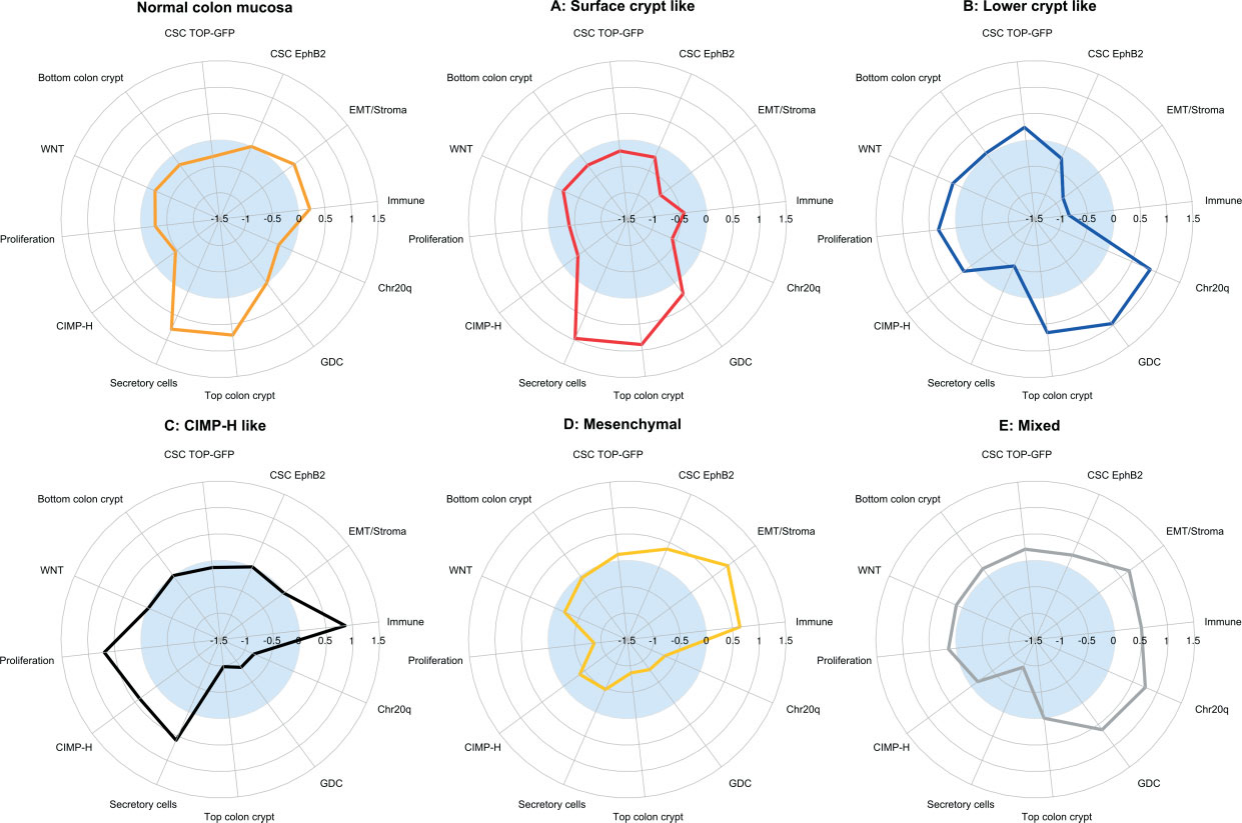
Subtypes and biological motifs. Subtype-specific fingerprints of biological motifs, represented either as mean values of gene set enrichment scores of gene sets from corresponding gene modules (EMT/stroma, immune, secretory cells, proliferation, GDC, chromosome 20q, top of the crypt—meta105 and meta144) or composed gene set enrichment scores of particular signatures (canonical Wnt targets, CSC-TopGFP, CSC-EphB2, colon crypt bottom and CIMP-H). The gene set enrichment scores represent whether the genes from the gene set show statistically significant enrichment between the down-regulated (negative scores, light blue area) or up regulated (positive scores) genes of a given subtype; details of score calculation can be found in the Supplementary material (Supplementary methods and results and Table S7.).

We investigated the association of subtypes with Wnt 28—32, putative colon cancer stem cell (CSC) 33,34 signatures, and two signatures specific for upper and lower colon crypt compartments 36, using gene set enrichment analysis (Figure 2; see also Supplementary material, Table S7). Subtypes B and E highly expressed canonical Wnt signalling target signatures. Subtypes A and D and also normal samples, however, showed low expression of these signatures. This was in concordance with the differences in *β*-catenin nuclear immunoreactivity at the invasion front (IF; see Supplementary material, Figure S9 and Supplementary methods and results). Subtypes B and E showed the highest percentages, while subtypes A and D showed significantly lower percentages of the *β*-catenin-positive nuclei. Subtype C exhibited almost no *β*-catenin nuclear immunoreactivity at the IF. We analysed CSC signatures derived from low colon crypt compartment cells that had been identified either by a Wnt reporter construct TOP GFP or by high surface expression of *EphB2*. Subtypes D and E expressed both TOP GFP and *EphB2*-derived CSC signatures, while subtype B mainly expressed only the TOP GFP signature (Figure 2).

### Subtypes complement clinical and molecular markers

**Figure 3:**
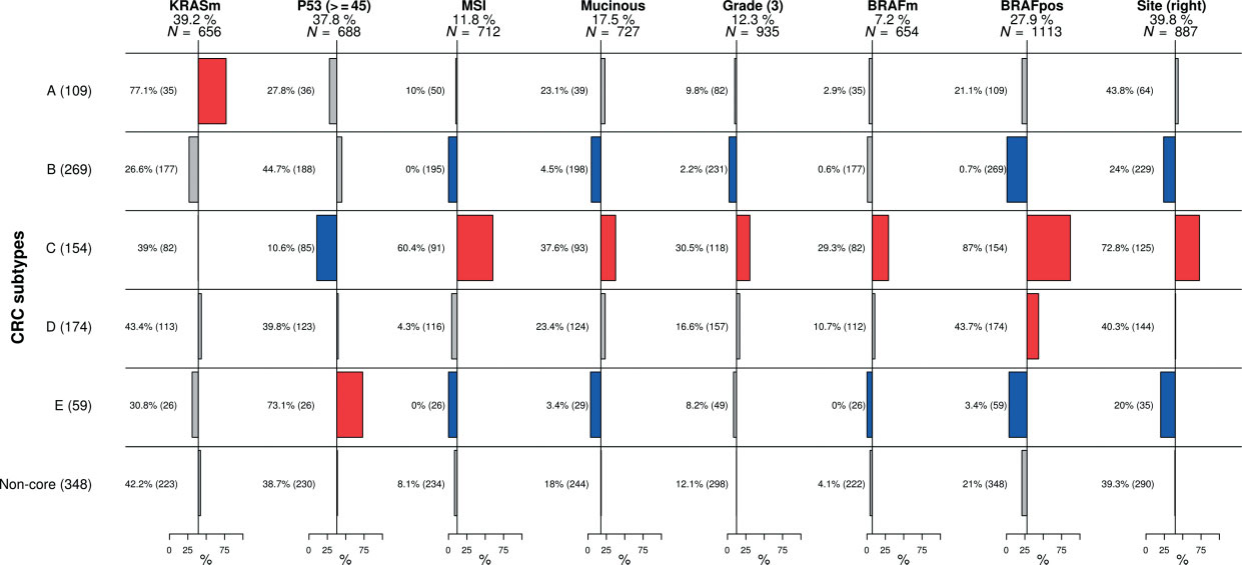
Clinical and mutational characterization of subtypes. Columns represent variables and rows subtypes. Horizontal bar plots represent proportions of the corresponding variable in each of the subtypes and non-core samples. Non-core samples were tested as one group to ensure that they did not share a common characteristic that would set them apart. Numbers in brackets adjacent to subtype name represent overall number of samples in the subtype. Under the title of each variable we denote the percentage representing baseline proportion in the population, with available information, and *N* denotes the number of patients for which the information on the respective feature was available. Bars in red represent significant enrichment and bars in blue significant depletion of a feature in the subtype in comparison to baseline, at the 5% significance level. Adjacent to each bar is the percentage of samples in the subtype with the specific feature and in brackets the overall number of samples in the subtype with the information available. We can read that, for instance, subtype C, comprising 154 samples, is enriched for microsatellite-unstable (MSI) tumours, where 60.4% of 91 samples with available information are MSI.

An important goal of this study was to assess how our molecular subtypes complement known clinical variables and molecular markers. We found that MSI, *BRAF* mutation status, site, mucinous histology and expression of p53 were significantly associated with various subtypes (Figure 3), but not tumour stage, age, gender, *SMAD4* or *PIK3CA* mutations (see Supplementary material, Figure S5A). Subtype D was not significantly enriched for any of the tested variables except for the *BRAF* mutated signature and possibly represents a mixture of tumours that have the EMT/stroma signature in common. *KRAS* mutants occurred in all subtypes (see Supplementary material, Figure S5C), supporting the emerging notion that *KRAS*-mutated CRC are substantially heterogeneous 5,6, the oncogenic role of *KRAS* varying per specific mutation and the molecular background of the tumour in which it occurs 38. Subtype C expressed the *BRAF* mutant signature we identified earlier 6 (87.0%), a CIMP-H signature (11, Figure 2), and its characteristics (enrichment for MSI, right side and mucinous histology) corresponded with those of the previously reported CIMP-H phenotype 9—40 and hypermutated tumours 13. Regarding the latter, subtype C had a similar low frequency of copy number variations (see Supplementary material, Figure S7). The distribution of MSI status, stage, age, gender, grade and site over the subtypes in the validation set followed the same patterns established in the discovery set [cf Figures 3 and S5B (see Supplementary material)]. A classification tree, trained with a combination of available clinical and molecular markers, did not identify our subtypes (see Supplementary material, Figure S5D), indicating that gene expression patterns reveal a layer of heterogeneity that goes beyond conventional CRC classification approaches.

### Histological characteristics of subtypes

To study whether or not our molecular subtypes are associated with histological patterns, we examined haematoxylin and eosin (H&E)-stained paraffin sections of a randomly selected subset of each subtype (23, 31, 31, 29 and 19 cases for subtypes A, B, C, D and E, respectively). In attempting to match histological morphotypes to molecular subtypes, architectural patterns were used, as illustrated in Figure 4A, rather than the recognized WHO classification of CRCs 1. Not surprisingly, given intratumour heterogeneity, none of the tumours had a single pattern. However, the prevalent patterns showed appreciable differences between the subgroups (Figure 4B, C; see also Supplementary material, Figure S6). In subtype A, the serrated pattern was most frequent, followed by the papillary pattern; in subtypes B and E, complex tubular dominated; in subtype C the solid pattern dominated, with mucinous as the second; most striking was the presence of a strong stromal reaction in subtype D.

## Discussion

Our approach, using gene modules on a large panel of samples, allowed us to identify five main CRC gene expression subtypes (Table 4). It is relevant to note that subtyping can be performed on FFPE tissues, an important prerequisite for wide clinical applications. An example is the hypermutated group identified in the TCGA study by whole exome sequencing 13, but according to our data also by gene expression profiling on routinely processed tissues (CIMP-H-like subtype).

**Table 4:** Summary of subtype characteristics

Subtype	CRC markers and mutations					Histopathology	IHC	Median survival (months)										Clinical												Gene expression													
	MSI	*BRAF*	*KRAS*	*P53*	Dominant	Nuclear *β*-catenin at IF	OS	RFS	SAR	Site	Grade	Up-regulated	Down-regulated
A: Surface crypt-like		–	+		Papillary or serrated	–	NA	NA	28.9			Top colon crypt, secretory cell, metallothioneins	EMT/stroma, Wnt, CSC, Chr20q, proliferation
B: Lower crypt-like	–	–			Complex tubular	+	NA	NA	50.4	Left	2	Top colon crypt, proliferation, Wnt	EMT/stroma, immune, secretory cell
C: CIMP-H-like	+	+		–	Solid/trabecular or mucinous	–	NA	NA	6.9	Right	3	Proliferation, immune, metallothioneins	GDC, top colon crypt, Chr20q
D: Mesenchymal					Desmoplastic	–	NA	79.5	19.8			EMT/stroma, CSC, immune	Proliferation, secretory cell, top colon crypt, GDC, Wnt, Chr20q
E: Mixed	–	–		+	Complex tubular	+	NA	NA	19.6	Left		EMT/stroma, immune, top colon crypt, Chr20q, GDC, CSC	Secretory cell

+, significantly enriched; –, significantly depleted; IF, invasion front; NA, not attained; no value, no significant enrichment in comparison to population baseline.

The combination of gene expression, clinical, mutational, survival and morphological data contributes new insight into the heterogeneity of CRC. While the validation confirmed the robustness of our findings across different platforms (ALMAC versus Affymetrix), sample preparation methods (FFPE versus fresh-frozen) and dataset collections, larger datasets are necessary to assess and characterize the relevance of lower frequency subtypes (eg F, or further segregation of B into B1 and B2). Our data indicate that several major biological processes are key determinants of a complex subtype structure of CRC. Therefore our subtypes defined by gene expression do not substitute but complement groups defined by current clinicopathological variables and molecular markers. Notably, morphological subclassification of CRC has clearly reached its limits, given the often striking intratumour heterogeneity, which made us use a (primary and secondary) architectural pattern approach rather than the canonized histological subtypes (WHO). Profiling of microdissected patterns within a single tumour might reveal molecular mechanisms responsible for these morphotypes. This additional heterogeneity within the subtypes may reflect tumour polyclonality, similar to breast cancer 41. Ultimately, aggregating clinical, pathological and further detailed molecular characteristics (including CNV, miRNA and methylation) will contribute to a more detailed perception of CRC heterogeneity and it is likely that more subtypes will emerge. This, however, would need more detailed molecular annotation of larger clinically well documented CRCs.

A striking association was found between the stromal subtype D and the EMT signature. The previously discovered EMT 12 also emerged from our analysis as the largest cluster of meta-genes associated with poor RFS (subtype D). Our histological assessment suggests that the EMT signature is the reflection of a strong mesenchymal stromal reaction, and this histological characteristic deserves to be tested for its capacity to predict resistance to therapy, in view of its strong association with poor survival. Studies requiring high tumour cell content as sample inclusion criteria (eg 13) could miss this poor prognosis subtype. Identification of this subtype in cell lines or xenograft models is less straightforward and would benefit from the analysis of gene expression patterns between microdissected tumour and stromal cells.

EMT, however important, only partly explains CRC heterogeneity, as even subtypes with similar expression of EMT-associated genes (A–C or D–E) differ in survival, mutational, clinical and gene expression characteristics. Additional biological components, such as differentiation, immune response, proliferation, chromosome 20q or cluster of genes deregulated in CRCs, are important co-determinants that underpin a need for further subdivision of CRCs. The findings from the analysis of CSC and WNT signatures support the recently suggested hypothesis that the colon stem cell signature under the condition of silenced canonical WNT targets is associated with higher risk of recurrence (subtype D) 33. This is consistent with subtype D showing a significantly lower percentage of *β*-catenin-positive nuclei than subtype B, with its Wnt-associated gene expression and better survival.

MSI tumours represent a subclass in most unsupervised analyses and can be recognized at the gene expression level 42. The more recent gene expression studies 14—15 suggest that MSI and *BRAF* share distinct gene expression patterns. Subtype C was enriched for both MSI and *BRAF* mutants and had one of the best outcomes for RFS, but the worse outcome in SAR, in concordance with previously reported results 43. Subtype C retained its poor SAR prognostic value, even in the population of MSS and *BRAF* wild-type patients. Our data suggest that subtype C represents tumours with a common biology and a gene expression pattern that might best characterize a group of tumours resistant to chemotherapy, once metastatic. In this sense, our work not only agrees with the current known markers (*BRAF* mutation status and MSI) but clearly adds new insight, putting together these previously unrelated clusters into one biologically meaningful group. This observation is in line with recently published work 6.

Our observations show that gene expression profiling contributes substantially to our insight into CRC heterogeneity in confirming and complementing data from sequencing, CNV and promoter methylation analysis. Our subtypes can be further functionally interrogated for driving oncogenes/events by *in vitro* functional screens. High-risk subtypes D and C might contribute to therapeutic decision making in either adjuvant or metastatic settings. Retrospective analysis of clinical trial series may identify drug sensitivity associated with particular subtypes, and might open new treatment optimization strategies to be tested in clinical trials with stratified cohorts, similar to the I-SPY2 trial for breast cancer 44.

In conclusion, our unsupervised approach using gene modules resulted in the identification of distinct molecularly defined CRC subtypes, which adds a new layer of complexity to CRC heterogeneity and opens new opportunities for understanding the disease. The challenge is now to assimilate conventional and these new molecular approaches into a comprehensive consensus classification, which might then be used in further clinical studies for patient stratification and experimental studies to further elucidate mechanisms involved in the development and progression of CRC.

## Supplementary material on the internet

The following supplementary material may be found in the online version of this article:

Supplementary methods and results (contains a further table and two further figures)

Figure S1 (A) Consensus clustering and similarity dendrogram of samples. (B) Subtype projection in the four-dimensional space of LDA axes. (C) Heat map matrix of pairwise meta-gene Fisher Z-transformed Pearson pairwise correlations. (D) Box plots of intra gene module pairwise gene–gene Pearson correlations in normal samples in both discovery and validation sets

Figure S2 Validation of meta-gene expression pattern of subtypes represented by heat maps

Figure S3 (A) Heat map representing validation of gene expression patterns of subtypes. (B) Pairwise Fisher Z-transformed correlations of meta-genes in validation set. (C) Box plots representing medians of pairwise gene–gene Pearson correlations in the validation datasets

Figure S4 Expression of top five down- and top five u*p* regulated genes from all pairwise comparisons between subtypes

Figure S5 (A) Other clinical and mutational markers tested and found non-significant between subtypes. (B) Clinical variables tested in the clusters of the validation test. (C) Distribution of significant clinical and mutational markers across subtypes. (D) Classification tree trained on clinical variables

Figure S6 Graphs of joined distribution of dominant vsersus secondary patterns in each of the subtypes

Figure S7 Heat map of CNV profiles of 154 samples from the discovery set, randomly ordered inside each of the subtypes

Figure S8 Result of hypothesis testing of median log-scale copy number estimates of chromosome 20 of subtype B versus all other subtypes

Figure S9 Distribution of *β*-catenin immunoreactivity of the invasion front counts between subtypes

Table S1 Detailed description of gene module members and detailed results of meta-gene expression tests pairwise between subtypes and of subtypes to meta-gene medians

Table S2 Multiclass linear discriminant (LDA) subtype assignment of samples from validation set

Table S3 Correlations of subtype-specific gene expression profiles (1 versus all moderated *t* test statistics) when accounting for subtype F in the training set

Table S4 Detailed results of meta-gene expression tests pairwise between subtypes and of subtypes to meta-gene medians

Table S5 Detailed results of pairwise comparisons of differentially expressed gene between subtypes

Table S6 Detailed results of Cox proportional hazards models for RFS, OS and SAR for subtype, stage, MSI and *BRAF* and for meta-genes

Table S7 Results of GSEA comparison of enrichment tested signatures in individual subtypes and normal samples
