# Supplementary figures and images for: Gene expression patterns unveil a new level of molecular heterogeneity in colorectal cancer
Source: J Pathol. 2013 Jul 8;231(1):63–76. doi: 10.1002/path.4212 (PMC3840702; doi:10.1002/path.4212)

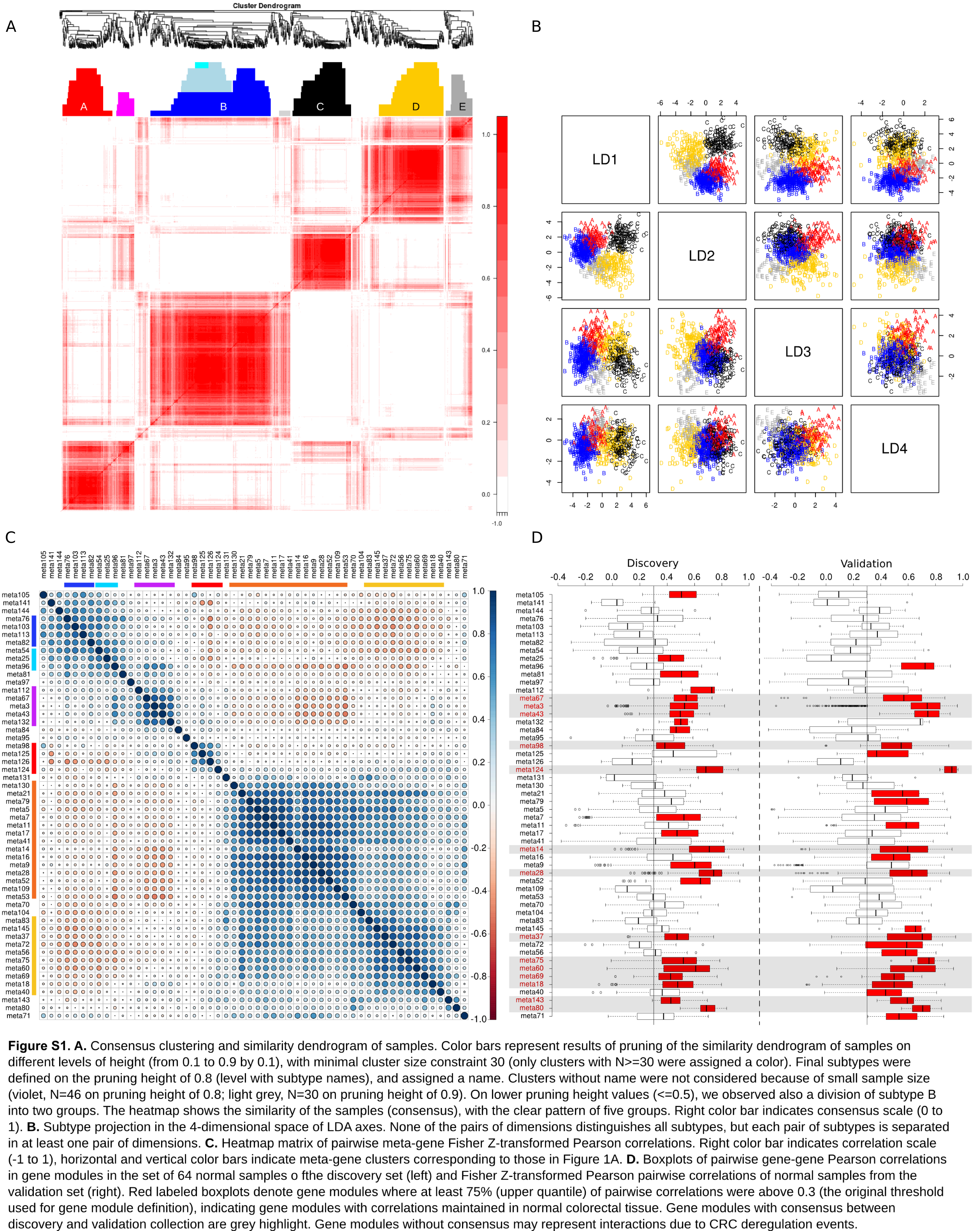

Supplement: Figure S1 — (A) Consensus clustering and similarity dendrogram of samples. (B) Subtype projection in the four-dimensional space of LDA axes. (C) Heat map matrix of pairwise meta-gene Fisher Z-transformed Pearson pairwise correlations. (D) Box plots of intra gene module pairwise gene–gene Pearson correlations in normal samples in both discovery and validation sets [file path0231-0063-sd1.tiff]

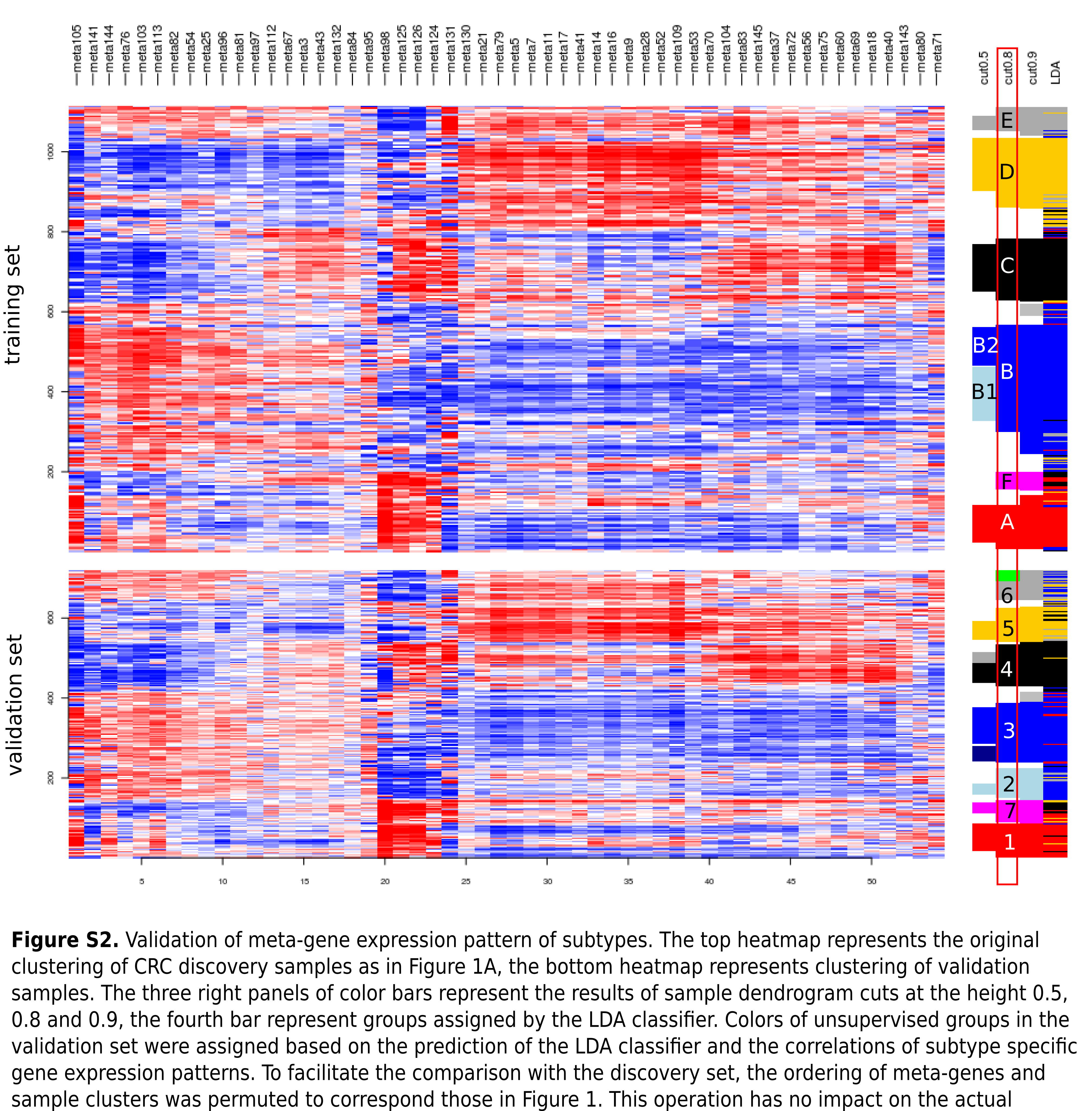

Supplement: Figure S2 — Validation of meta-gene expression pattern of subtypes represented by heat maps [file path0231-0063-sd2.tiff]

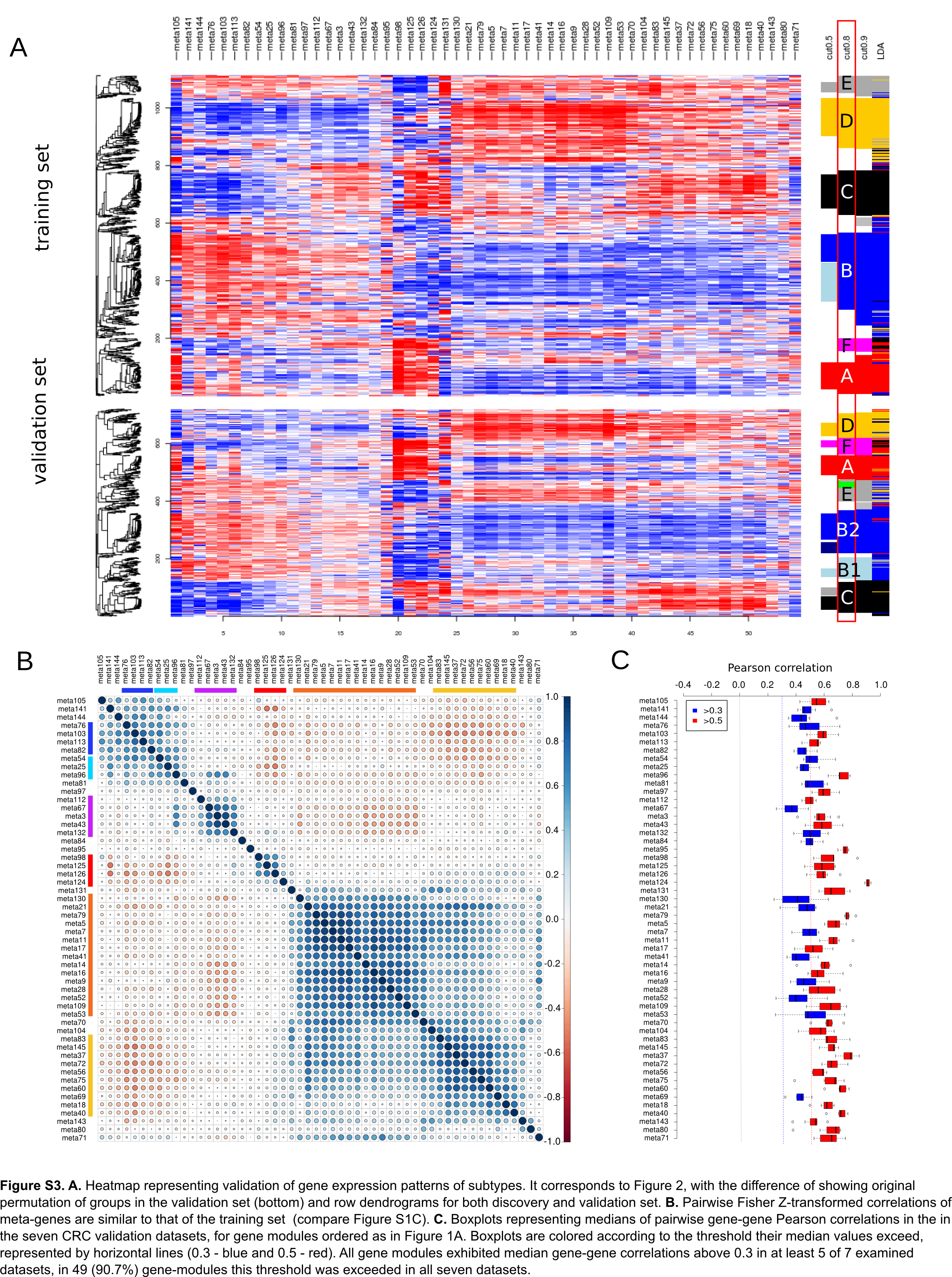

Supplement: Figure S3 — (A) Heat map representing validation of gene expression patterns of subtypes. (B) Pairwise Fisher Z-transformed correlations of meta-genes in validation set. (C) Box plots representing medians of pairwise gene–gene Pearson correlations in the validation datasets [file path0231-0063-sd3.tiff]

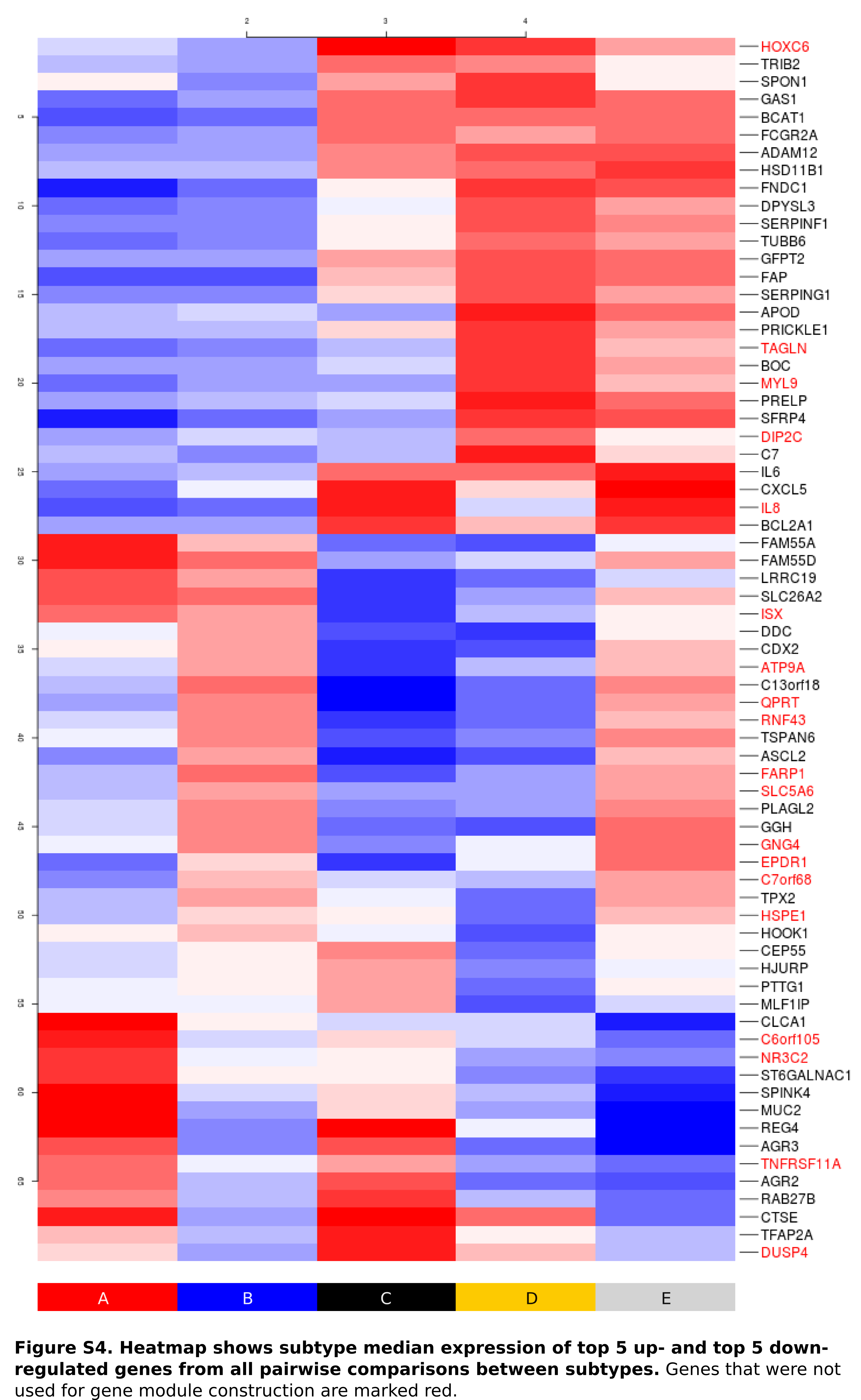

Supplement: Figure S4 — Expression of top five down- and top five up regulated genes from all pairwise comparisons between subtypes [file path0231-0063-sd4.tiff]

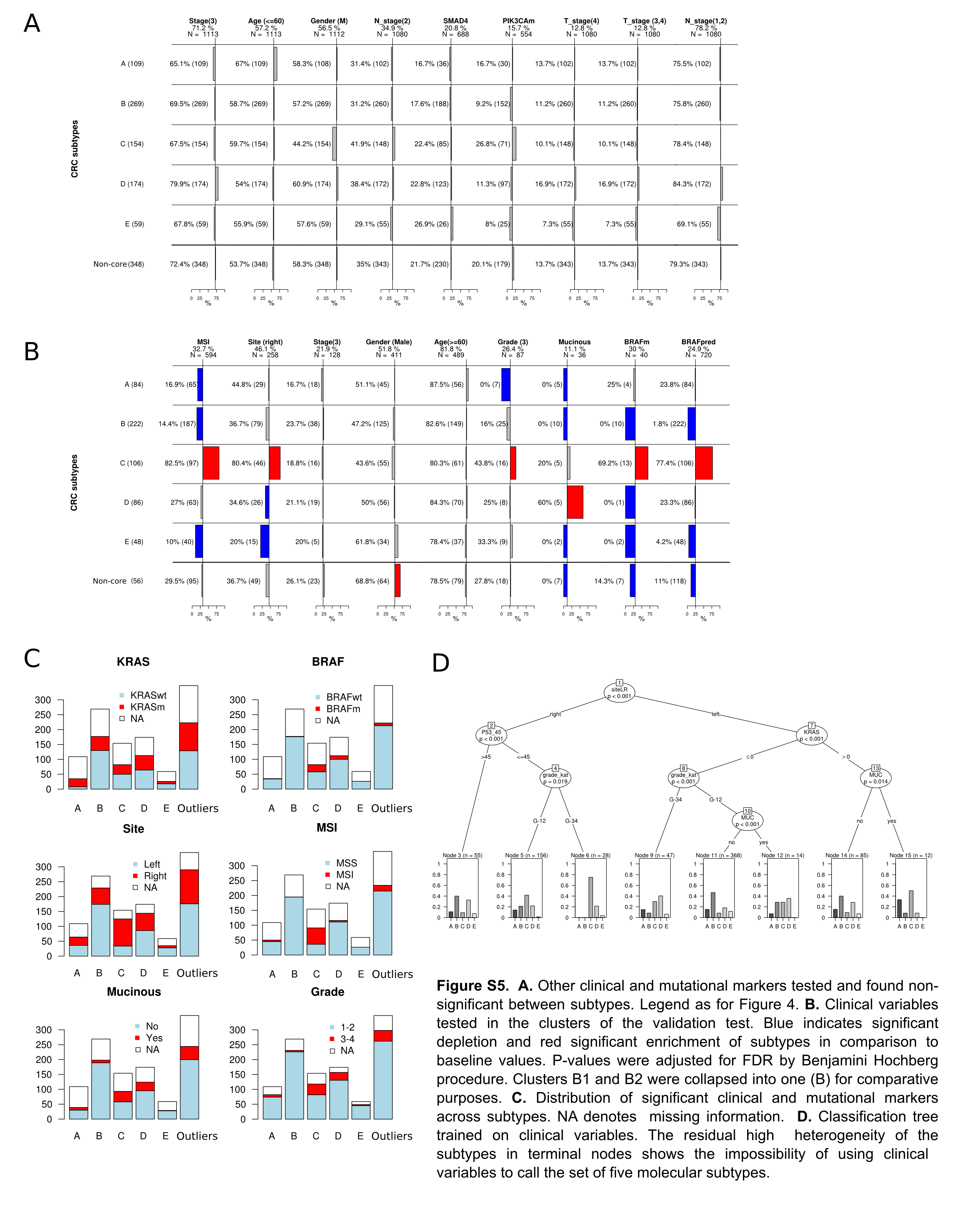

Supplement: Figure S5 — (A) Other clinical and mutational markers tested and found non-significant between subtypes. (B) Clinical variables tested in the clusters of the validation test. (C) Distribution of significant clinical and mutational markers across subtypes. (D) Classification tree trained on clinical variables [file path0231-0063-sd5.tiff]

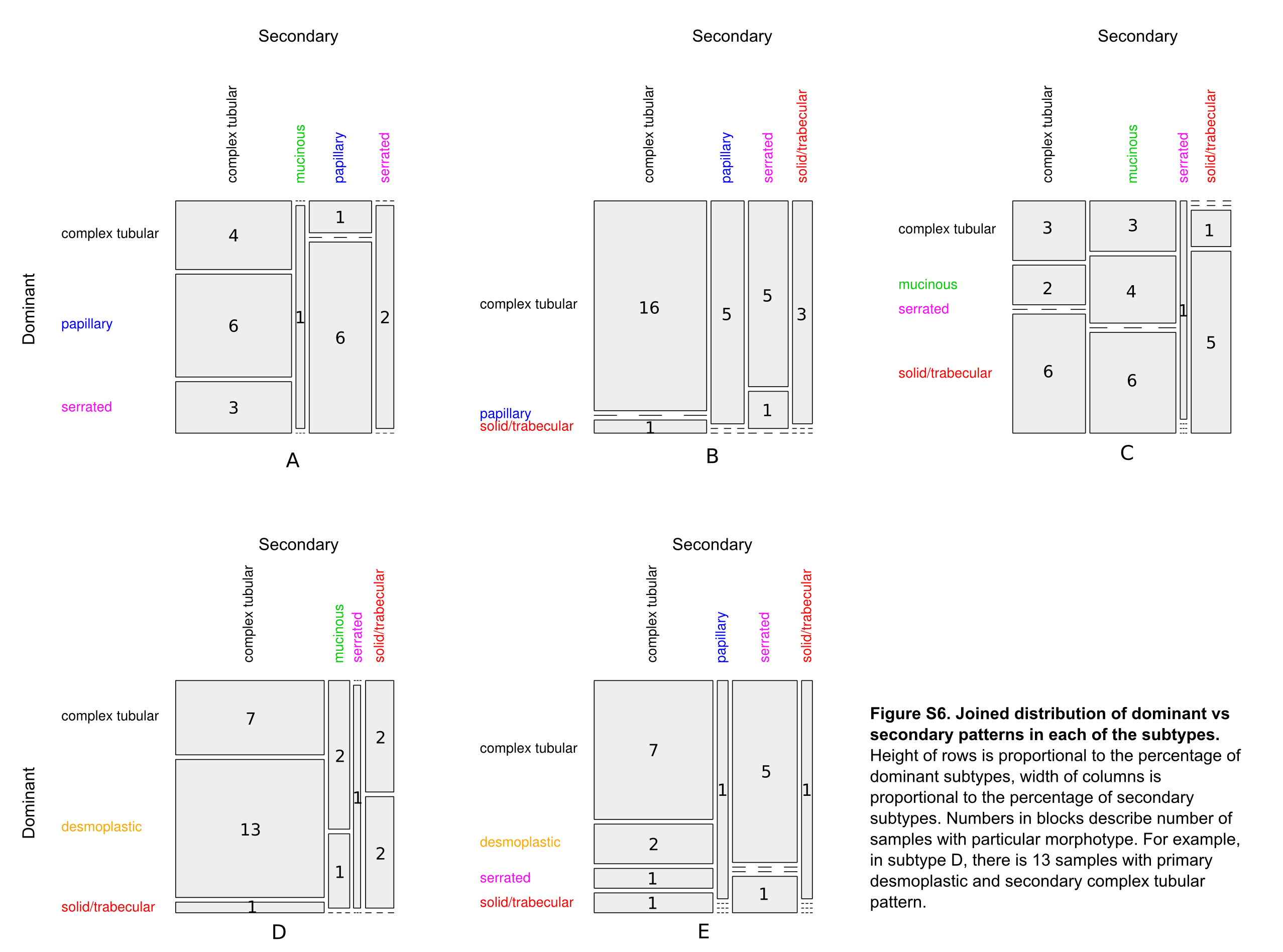

Supplement: Figure S6 — Graphs of joined distribution of dominant vsersus secondary patterns in each of the subtypes [file path0231-0063-sd6.tiff]

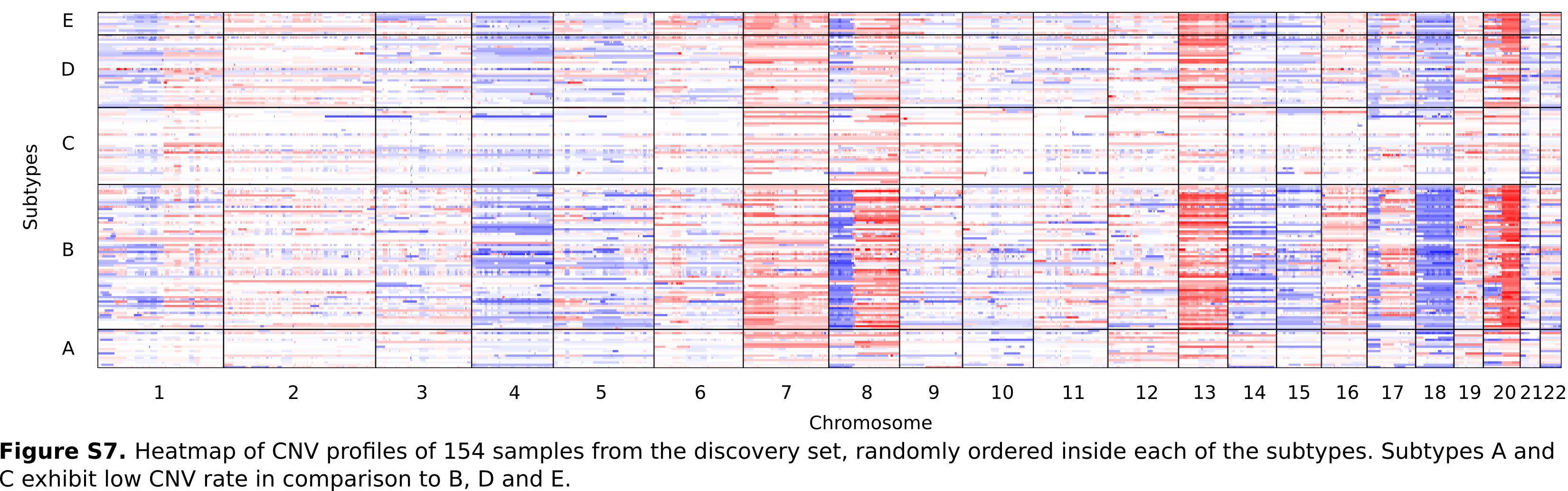

Supplement: Figure S7 — Heat map of CNV profiles of 154 samples from the discovery set, randomly ordered inside each of the subtypes [file path0231-0063-sd7.tiff]

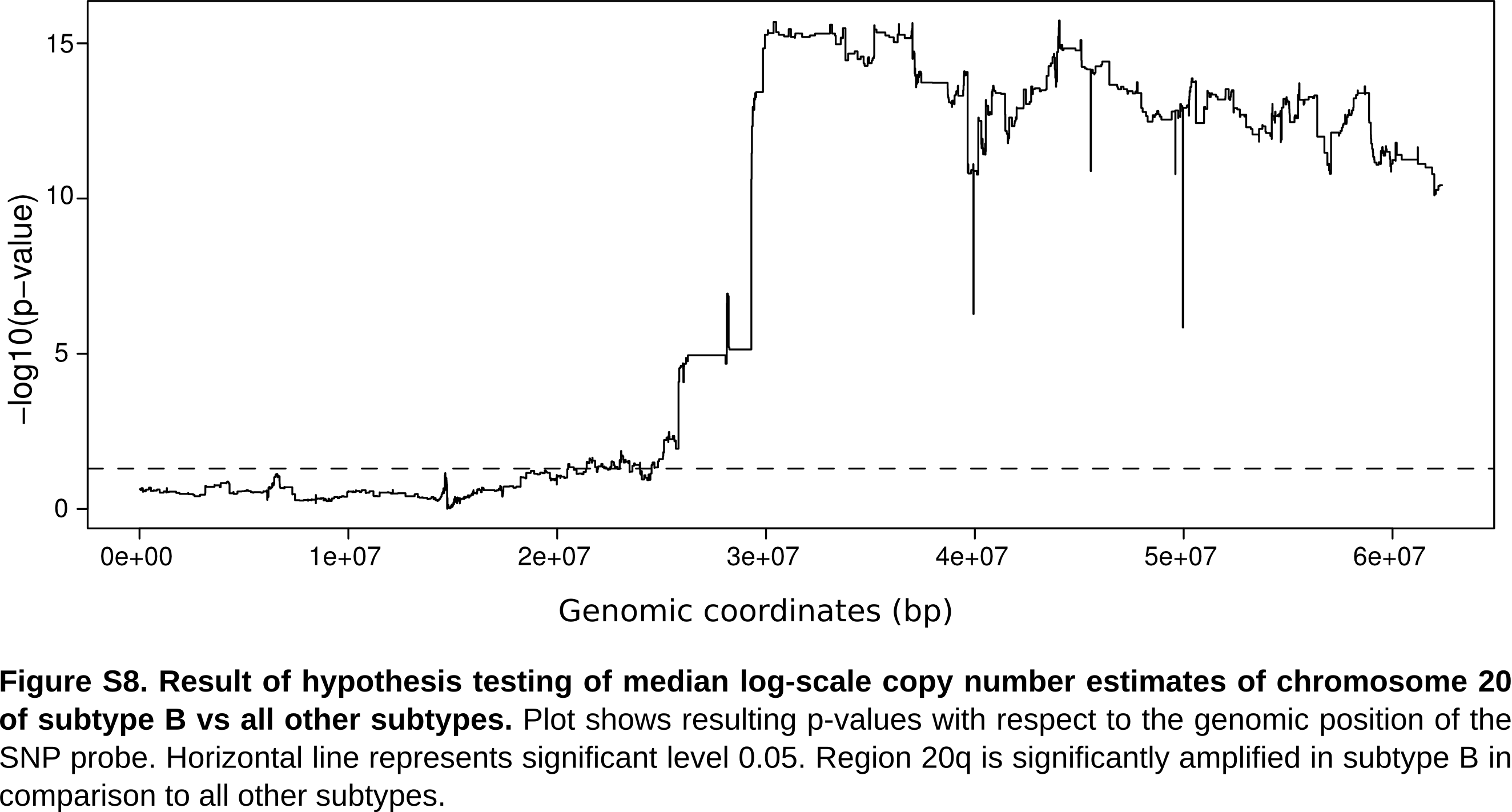

Supplement: Figure S8 — Result of hypothesis testing of median log-scale copy number estimates of chromosome 20 of subtype B versus all other subtypes [file path0231-0063-sd8.tiff]

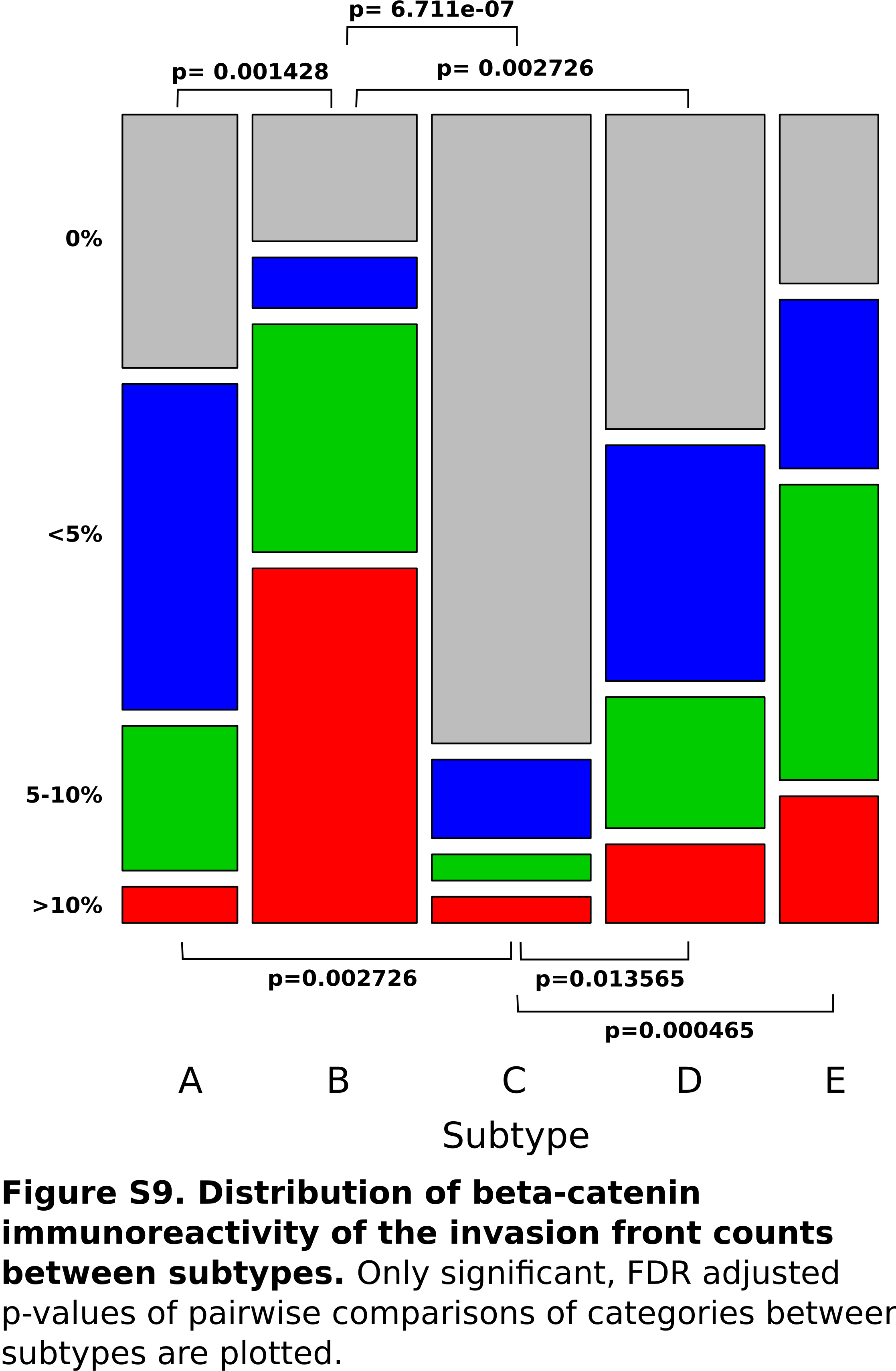

Supplement: Figure S9 — Distribution of β-catenin immunoreactivity of the invasion front counts between subtypes [file path0231-0063-sd9.tiff]
