# Supplementary material for: Gene expression patterns unveil a new level of molecular heterogeneity in colorectal cancer
Source: J Pathol. 2013 Jul 8;231(1):63–76. doi: 10.1002/path.4212 (PMC3840702; doi:10.1002/path.4212)
Supplement: Table S2 — Multiclass linear discriminant (LDA) subtype assignment of samples from validation set [file path0231-0063-sd11.doc]

**Table S2.** Multiclass linear discriminant (LDA) subtype assignment of samples from validation set.

| **Validation cluster/ corresponding subtype**  **in discovery set** | **LDA assignment** | | | | | **SUM** |
| --- | --- | --- | --- | --- | --- | --- |
|  | **A** | **B** | **C** | **D** | **E** |  |
| **1/A** | **74** | 4 | 3 | 3 | 0 | 84 |
| **2/B1** | 1 | **58** | 0 | 2 | 13 | 74 |
| **3/ B2** | 12 | **134** | 1 | 0 | 1 | 148 |
| **4 / C** | 1 | 2 | **99** | 4 | 0 | 106 |
| **5 / D** | 0 | 3 | 12 | **64** | 7 | 86 |
| **6 / E** | 1 | 17 | 0 | **17** | **13** | 48 |
| **7 / F** | **23** | 1 | **22** | 9 | 1 | 56 |
| **Non-core** | 21 | 53 | 18 | 8 | 18 | 118 |
| **SUM** | 133 | 272 | 155 | 107 | 53 | **720** |
