# Supplementary material for: Gene expression patterns unveil a new level of molecular heterogeneity in colorectal cancer
Source: J Pathol. 2013 Jul 8;231(1):63–76. doi: 10.1002/path.4212 (PMC3840702; doi:10.1002/path.4212)
Supplement: Table S3 — Correlations of subtype-specific gene expression profiles (1 versus all moderated t test statistics) when accounting for subtype F in the training set [file path0231-0063-sd12.docx]

**Table S3.** Correlations of subtype specific gene expression profiles (1 vs all moderated t-test statistics), when accounting for subtype F in the training set.

|  | **Subtypes from training set most correlated to validation subtypes** | | | | | |
| --- | --- | --- | --- | --- | --- | --- |
|  | **First subtype** | | | **Second subtype** | | |
| **Validation cluster/ corresponding subtype**  **in discovery set** | **Subtype** | **Cor** | **P-val** | **Subtype** | **Cor** | **P-val** |
| **1 / A** | A | 0.85 | p<1.0E-15 | F | 0.41 | p<1.0E-15 |
| **2 / B1** | B | 0.71 | p<1.0E-15 | E | 0.47 | p<1.0E-15 |
| **3 / B2** | B | 0.91 | p<1.0E-15 | A | 0.36 | p<1.0E-15 |
| **4 / C** | C | 0.89 | p<1.0E-15 | F | 0.29 | p<1.0E-15 |
| **5 / D** | D | 0.93 | p<1.0E-15 | E | 0.37 | p<1.0E-15 |
| **6 / E** | E | 0.63 | p<1.0E-15 | D | 0.58 | p<1.0E-15 |
| **7 / F** | F | 0.61 | p<1.0E-15 | C | 0.55 | p<1.0E-15 |
